# Migraine and greater pain symptoms at 10-year follow-up among patients with major depressive disorder

**DOI:** 10.1186/s10194-018-0884-9

**Published:** 2018-07-17

**Authors:** Ching-I Hung, Chia-Yih Liu, Ching-Hui Yang, Shuu-Jiun Wang

**Affiliations:** 1 Department of Psychiatry, Chang-Gung Memorial Hospital at Linkou and Chang-Gung University College of Medicine, Tao-Yuan, Taiwan; 2 Department of Nursing, Chang Gung University of Science and Technology, Tao-Yuan, Taiwan; 3 Faculty of Medicine and Brain Research Center, National Yang-Ming University and Neurological Institute, Taipei Veterans General Hospital, Taipei, Taiwan; 4 Department of Neurology, Taipei Veterans General Hospital, No. 201 Shi-Pai Road, Section 2, Taipei, 112 Taiwan

**Keywords:** Depression, Headache, Pain, Somatization, Quality of life

## Abstract

**Background:**

No study has investigated the associations of migraine with pain symptoms over a ten-year period among outpatients with major depressive disorder (MDD). This study aimed to investigate this issue.

**Methods:**

At baseline, the study enrolled 290 outpatients with MDD and followed-up the patients at six-month, two-year, and ten-year time points. MDD and anxiety comorbidities were diagnosed using the Structured Clinical Interview for DSM-IV-text revision. Migraine was diagnosed based on the International Classification of Headache Disorders. The bodily pain subscale of the Short Form 36 (SF-BP) and the pain subscale (PS) of the Depression and Somatic Symptoms scale were also used. Generalized Estimating Equation models were employed to investigate the longitudinal impacts of migraine on pain symptoms.

**Results:**

MDD patients with migraine had lower SF-BP and higher PS scores than those without. Depression, anxiety, and headache indices were significantly correlated with SF-BP and PS scores. The higher the frequency of migraine, the more often patients suffered from pain symptoms. Patients with migraine at all investigated time points suffered from pain symptoms most of the time (ranging from 60.0% to 73.7%) over the 10 years. After controlling for depression and anxiety, migraine was independently associated with a decreased SF-BP score (by 8.93 points) and an increased PS score (by 1.33 points).

**Conclusion:**

Migraine was an important comorbidity associated with greater severities of pain symptoms during long-term follow-up. Migraine treatment should be integrated into the treatment of depression to improve pain symptoms and quality of life in the pain dimension.

## Background

Painful physical symptoms and depression interact with each other [1, 2]. Major depressive disorder (MDD) and pain symptoms may have a shared neurobiological mechanism and may be genetically correlated [3, 4]. Among patients with MDD, pain symptoms are common, and are associated with a greater severity of depression, a poorer treatment response, inability to achieve full remission, impaired function, a poorer quality of life, and an increased suicidal risk [3, 5–9].

MDD, migraine, anxiety, and pain symptoms mutually affect each other [10–12]. For example, strong bidirectional associations have been suggested to exist between psychiatric disorders, migraine, and suicide [13]. Among patients with chronic migraine, a higher affective dysregulated temperament score was found to be associated with a greater feeling of hopelessness and a higher suicidal risk [13]. Shared underlying genetic mechanisms have been reported for MDD and migraine [14]. In addition, migraine is also associated with increased risks of other diseases, such as cardiovascular diseases, cerebrovascular diseases, and fibromyalgia [15–19].

Nearly half of patients with MDD have comorbid migraine [20, 21]. MDD patients with migraine have greater severities of depression, anxiety, and pain symptoms than those without migraine [21, 22]. Migraine also has negative impacts on the recovery of health-related quality of life (HRQoL) and some pain symptoms after acute treatment [22, 23]. However, most of these studies were cross-sectional or acute treatment studies, and few studies have investigated the impacts of migraine on MDD during a long-term follow-up period. In a two-year follow-up study, migraine at baseline was an independent factor associated with upper and lower limb muscle soreness at the two-year follow-up point [24]. Another study found that migraine with active headache at follow-up was associated with greater severities of anxiety and somatic symptoms [25].

Several studies have investigated the associations of migraine with pain symptoms [22–24]. However, to our knowledge, no study has investigated the associations of migraine with the pain dimension of the HRQoL and pain symptoms among patients with MDD during a ten-year period. This issue is important for the following reasons: 1) pain symptoms are common, and are associated with a poorer prognosis of MDD [3, 7]; 2) migraine is a common comorbidity of MDD [20, 21], and the associations of migraine with pain symptoms should be clarified; and 3) in some patients with MDD, depression and pain symptoms are chronic and fluctuating. Several factors have been identified to be associated with chronic depression, such as a younger age at onset, a longer duration of depressive episode, a family history of mood disorders, and psychiatric comorbidities, including anxiety disorders, personality disorders, and substance abuse [26]. Factors associated with persisting pain symptoms post-three-month treatment among patients with MDD include a greater depressive severity, age < 40 years, more than one comorbidity, and previous MDD episodes [8]. In addition, persisting pain symptoms post-treatment were found to be associated with a poorer remission rate of depression [8]. Therefore, the outcomes of depression and pain symptoms interact and are correlated. Long-term follow-up studies are mandatory. Therefore, this study aimed to investigate the associations of migraine with the pain dimension of the HRQoL and pain symptoms among patients with MDD during a ten-year period. We hypothesized that migraine was associated with more severe pain symptoms and a poorer score in the pain dimension of the HRQoL among patients with MDD during a ten-year period, because migraine does not only encompass headache, but is also associated with other pain symptoms.

## Methods

### Subjects

This study was performed in the psychiatric outpatient clinics of the Chang Gung Memorial Hospital at Linkou, a medical center in northern Taiwan. At baseline (January 2004 to August 2007), consecutive outpatients who fulfilled the following three criteria were considered eligible subjects: 1) 18−65 years of age; 2) no antidepressants or other psychotropic drugs administered within the previous four weeks; and 3) met the DSM-IV-TR criteria for MDD and were experiencing a current major depressive episode (MDE) [27]. MDD and anxiety disorders were diagnosed according to the Structured Clinical Interview for DSM-IV-TR Axis I Disorders [28]. Moreover, three exclusion criteria were established to prevent confounding of somatic and pain symptoms, including 1) catatonic features, psychotic symptoms or severe psychomotor retardation with obvious difficulty in being interviewed; 2) a history of substance abuse or dependence without full remission in the previous month; and 3) chronic medical diseases such as hypertension, diabetes mellitus, and other medical diseases, except for headache.

At baseline, 290 subjects were enrolled. These patients were followed-up at the six-month, two-year, and ten-year points. The ten-year follow-ups were performed from August 2014 to December 2016. The study was approved by the Institutional Review Board of the Chang Gung Memorial Hospital. Based on the guidelines regulated in the Declaration of Helsinki, written informed consent was obtained from all subjects.

### Assessment of headache

At baseline, all subjects completed a structured headache intake form, which reported headache patterns over the past year and included headache intensity, frequency, location, duration, aggravation by physical activities, phonophobia, photophobia, nausea, vomiting, precipitating factors, painkiller use, and aura. Then, an experienced headache specialist, who was blind to other results, interviewed all subjects and made headache diagnoses. This procedure was also performed at the two-year and ten-year follow-up points. At the six-month follow-up, diagnosis of migraine was taken as the baseline diagnosis.

At baseline and the two-year follow-up point, headache was diagnosed based on the International Classification of Headache Disorders, 2nd edition (ICHD-2) [29]. At the ten-year follow-up point, headache was diagnosed based on the ICHD-3 beta, and headache diagnoses at baseline and the two-year follow-up point were also updated to ICHD-3 beta diagnoses [30].

At each time point, subjects who fulfilled the criteria of migraine without aura (MO) and/or migraine with aura (MA) were classified as the “migraine” group, while the other subjects were categorized as the “non-migraine” group. The intensity and frequency of headaches were assessed at baseline and the three follow-up points. We used the visual analog scale (VAS) to evaluate the average headache intensity in the past week, rated from 0 (no pain) to 10 (pain as severe as I can imagine), and recorded the frequency of headache days.

### Assessment of anxiety comorbidities

One psychiatrist, who was blind to other psychiatric data and headache diagnoses, used the Structured Clinical Interview for DSM-IV-TR Axis I Disorders to diagnose the following anxiety comorbidities: panic disorder, agoraphobia, specific phobia, social phobia, post-traumatic stress disorder, obsessive–compulsive disorder, and generalized anxiety disorder [28]. Subjects with any one of the seven anxiety disorders were classified as the “with any anxiety disorder” group, while the others were classified as the “without any anxiety disorder” group.

### Instruments for the evaluation of pain, depression, and anxiety

The bodily pain subscale of the Short Form-36 (SF-BP) and the pain subscale (PS) of the Depression and Somatic Symptoms Scale (DSSS) were used to evaluate the pain dimension of HRQoL and pain severities, respectively [31–35]. The acute version of the Short Form-36 was used. SF-BP scores ranged from 0 to 100, a higher score indicating a better HRQoL [32, 33]. The PS of the DSSS evaluated the severities of five pain symptoms (headache, back pain, chest pain, neck and/or shoulder pain, and general muscle pain) in the past week. The total score of the PS ranged from 0 to 15, a higher score indicating a greater severity of pain symptoms.

To understand the longitudinal course of pain symptoms in the past 10 years, subjects were requested to report the percentages of time during which they had suffered the following pain symptoms in the past 10 years: headache, back pain, shoulder and/or neck pain, and general muscle pain.

The Hamilton Depression Rating Scale (HAMD) and the anxiety subscale of the Hospital Anxiety and Depression Scale (HADS-A) were used to evaluate depression and anxiety, respectively [35–37]. The scores ranged from 0 to 52 and 0–21 for the HAMD and HADS-A, respectively, and higher scores indicated a greater severity of symptoms.

The native language of our subjects was Chinese. The reliability and validity of the Chinese versions of the three administered scales (SF-36, HADS, and DSSS) have been established [31, 33–35].

### Procedures

Subjects were followed-up at the time points of 6 months, 2 years, and 10 years after baseline enrollment. The SF-BP, PS, and HADS-A were administered at the three follow-up points, and the HAMD was evaluated by the same psychiatrist.

During the ten-year period, subjects might have quit pharmacotherapy or accepted pharmacotherapy intermittently. Pharmacotherapy was not controlled at the three follow-up points. At the index month of the follow-up point, patients who were not receiving pharmacotherapy were classified into the “without pharmacotherapy (in the index month)” group, whereas patients who were receiving pharmacotherapy were classified as the “with pharmacotherapy (in the index month)” group. This classification was used to avoid the confounding effects of pharmacotherapy in statistical analysis.

### Statistical methods

All statistical analyses were performed using SPSS for Windows 20.0. The independent *t* test, Mann-Whitney U test, Kruskal-Wallis H test, Pearson’s correlation, Spearman’s correlation, and Chi-square test were used in appropriate situations.

Generalized Estimating Equation (GEE) models with robust error estimation and an unstructured covariance matrix were used to estimate the differences in SF-BP and PS scores between patients with and without migraine. The dependent variables were the SF-BP and PS scores. Initially, 10 variables were placed into the GEE model as independent variables, including six variables at baseline (age, gender, marital status, educational years, employment, with any anxiety disorders or not) and four variables at each follow-up point (with migraine or not, with pharmacotherapy or not, HAMD score, HADS-A score). Then, insignificant factors were removed from the GEE model one by one until all independent factors were significant.

A two-tailed *P* value of < 0.05 was taken to indicate statistical significance, and Bonferroni correction was used in appropriate situations.

## Results

### Subjects

For clarity, the labels “(B)”, “(6 M)”, “(2Y)” and “(10Y)” are used to represent data collected at baseline, and at the six-month, two-year, and ten-year follow-up points, respectively. At baseline, 290 subjects were enrolled. Table 1 shows the demographic variables, percentages of subjects with comorbidities, pain indices, and psychometric scores. There were no significant differences between patients who were and were not followed-up at the three follow-up points in terms of the five demographic variables, with the exception of the variable of age at the ten-year follow-up point (with vs. without follow-up: 41.3 ± 8.1 vs. 39.3 ± 8.2 years, *p* = 0.04). At the six-month and two-year time points, the percentage of subjects who attended follow-up was over 80%. At the ten-year follow-up point, approximately half of the subjects attended follow-up appointments. Of the subjects who did not complete the ten-year follow-up assessment (*n* = 153), 34.1% (*n* = 99) were unable to be contacted by phone or mail; 16.9% (*n* = 49) refused to participate in the follow-up; 1.0% (*n* = 3) had expired due to medical diseases; and 0.7% (*n* = 2) had their psychiatric diagnosis shifted to schizophrenia.

**Table 1:** Demographic variables, pain indices, and psychometric scores at different time points among patients with major depressive disorder

Time point	Baseline	Six-month follow-up	Two-year follow-up	Ten-year follow-up
Case number	290	254	237	137
Percentage of loss follow-up	−	12.4	18.3	52.8
Age (years)	30.2 ± 8.2	30.6 ± 8.2	32.5 ± 8.4	41.0 ± 8.1
Educational years	13.2 ± 2.4	13.3 ± 2.4	13.3 ± 2.4	13.3 ± 2.5
Female (%)	71.4	70.9	69.2	69.3
In employment (%)	57.2	56.7	55.7	57.7
Married (%)	42.1	42.1	43.0	44.5
With migraine (%)	46.9	46.5	22.8	38.0
With any anxiety disorders (%)	51.4	50.4	54.4	49.6
With pharmacotherapy (%)	0	47.6	27.4	27.7
BMI	21.4 ± 4.0	−	−	23.8 ± 4.3
SF-BP scores	48.3 ± 22.9	65.5 ± 23.6	68.4 ± 21.8	65.3 ± 20.5
PS scores	7.6 ± 3.8	4.1 ± 3.6	4.2 ± 3.3	4.7 ± 3.0
HAMD scores	23.4 ± 4.2	10.6 ± 7.8	10.4 ± 7.4	9.4 ± 6.4
HADS-A scores	15.0 ± 3.3	8.6 ± 4.8	9.0 ± 4.5	8.4 ± 4.7

BMI: body mass index; HAMD: Hamilton Depression Rating scale; HADS-A: anxiety subscale of the Hospital Anxiety and Depression scale; SF-BP: the bodily pain subscale of the Short Form 36; PS: pain subscale of the Depression and Somatic Symptoms scale

### Headache and psychiatric diagnoses

Table 1 shows the percentages of patients with migraine at the different time points. At baseline, 136 subjects (46.9%) had migraine, including 116 MO (headache code 1.1) and 20 MO and MA (headache code 1.2). At the two-year follow-up point, 54 subjects (22.8%) had migraine, including 37 MO and 17 MO and MA. At the ten-year follow-up point, 52 subjects (38.0%) had migraine, including 42 MO and 10 MO and MA.

At baseline, 51.4% (*n* = 149) of the subjects had at least one anxiety disorder, including 12.1% (*n* = 35) with panic disorder, 11.7% (*n* = 34) with agoraphobia, 22.1% (*n* = 64) with specific phobia, 27.6% (*n* = 80) with social phobia, 10.7% (*n* = 31) with post-traumatic stress disorder, 9.3% (*n* = 27) with obsessive-compulsive disorder, and 5.5% (*n* = 16) with generalized anxiety disorder. Compared with the subjects without anxiety disorders, a higher percentage of the subjects with anxiety disorders had comorbid migraine (63.1% vs. 29.8%, *p* <  0.001).

Patients with migraine at the two-year and 10-year follow-up points had a longer total duration of pharmacotherapy (11.3 ± 8.1 vs. 7.8 ± 7.0 months, *p* = 0.002 at the two-year point; 28.3 ± 37.5 vs. 27.0 ± 35.8 months, *p* = 0.84 at the 10-year point) as compared with those without.

### Differences in pain indices between groups

Table 2 shows the differences in the SF-BP and PS scores between groups at the four time points. Patients with migraine had a lower SF-BP score and a higher PS score than patients without migraine at all four time points after Bonferroni correction, with the exception of the PS score _(2Y)_ in patients with pharmacotherapy.

**Table 2:** The differences of the pain indices at four time points between patients with and without migraine

			Without pharmacotherapy			With pharmacotherapy	
	Migraine	N	SF-BP	PS	N	SF-BP	PS
Baseline	Yes	136	37.7 ± 19.3**	9.6 ± 3.4**	−	−	−
Baseline	No	154	57.6 ± 21.8	5.9 ± 3.2	−	−	−
Six month	Yes	58	56.1 ± 26.9**	5.6 ± 4.1**	60	60.9 ± 21.8**	4.5 ± 3.9**
Six month	No	75	68.9 ± 22.6	3.8 ± 3.2	61	74.9 ± 18.9	2.5 ± 2.8
Two year	Yes	34	53.1 ± 22.2**	7.1 ± 4.3**	20	52.6 ± 18.7**	6.0 ± 2.7*
Two year	No	138	74.5 ± 19.9	3.1 ± 2.6	45	68.4 ± 19.8	4.4 ± 2.8
Ten year	Yes	38	59.0 ± 18.3**	5.9 ± 2.6**	14	47.3 ± 17.2**	7.3 ± 3.4**
Ten year	No	61	73.4 ± 19.5	3.6 ± 2.7	24	64.9 ± 19.0	4.1 ± 2.8

*SF-BP* the bodily pain subscale of the Short Form 36, *PS* the pain subscale of the Depression and Somatic Symptoms scale

*P <  0.05; ** Significance after Bonferroni correction with *P* <  0.025

### Differences in the severities of depression, anxiety and headache indices between groups

Patients with migraine had a higher headache intensity and frequency than patients without migraine after Bonferroni correction (Table 3). Patients with migraine also had greater severities of depression and anxiety after Bonferroni correction, with the exception of the severities of depression _(6M)_ and anxiety _(6M and 10Y)_ in patients without pharmacotherapy, and the severity of depression _(10Y)_ in patients with pharmacotherapy.

**Table 3:** The difference of psychometric scores and headache indices at four time points between patients with and without migraine^a^

			Without pharmacotherapy				With pharmacotherapy				
	Migraine	N	HAMD	HADS-A	HI	HF	N	HAMD	HADS-A	HI	HF
Baseline	Yes	136	24.8 ± 4.3**	15.7 ± 3.2**	6.4 ± 3.0**	4.3 ± 2.4**	−	−	−	−	−
Baseline	No	154	22.2 ± 3.8	14.3 ± 3.3	3.2 ± 2.7	2.3 ± 2.2	−	−	−	−	−
Six month	Yes	58	14.2 ± 9.1*	10.0 ± 5.0	3.7 ± 3.0**	2.8 ± 2.5**	60	10.5 ± 6.7**	9.1 ± 4.5**	2.9 ± 2.8**	2.2 ± 2.0***
Six month	No	75	11.0 ± 7.8	9.2 ± 4.7	1.5 ± 1.8	1.5 ± 2.2	61	6.7 ± 5.8	6.1 ± 4.1	1.4 ± 2.0	0.9 ± 1.4
Two year	Yes	34	16.3 ± 7.3**	12.0 ± 4.4**	5.2 ± 3.0**	2.6 ± 2.2**	20	15.7 ± 6.4**	11.9 ± 3.7**	4.8 ± 2.9**	2.6 ± 2.0**
Two year	No	138	8.4 ± 6.6	7.9 ± 4.1	1.5 ± 2.0	1.0 ± 1.5	45	9.6 ± 6.8	8.7 ± 4.3	2.3 ± 2.3	1.6 ± 1.8
Ten year	Yes	38	10.4 ± 5.5**	8.7 ± 4.8*	3.9 ± 3.0**	1.6 ± 1.5**	14	16.0 ± 6.8*	13.4 ± 4.5**	4.5 ± 3.5**	3.2 ± 2.7**
Ten year	No	61	6.4 ± 5.3	6.6 ± 4.1	1.3 ± 2.1	1.0 ± 1.5	24	11.3 ± 6.2	9.6 ± 3.7	1.3 ± 2.0	1.2 ± 1.7

*HAMD* hamilton depression rating scale, *HADS-A* anxiety subscale of the Hospital Anxiety and Depression scale, *HI* headache intensity, *HF* headache frequency

*P <  0.05; ** Significance after Bonferroni correction with *P* <  0.0125

^a^Headache intensity and frequency were measured using a 0–10 visual analog scale and the pain days of the past week, respectively

### Correlations of pain indices with depression, anxiety, and headache indices at the same time points

The SF-BP and PS scores were significantly (all *p* <  0.001) correlated with the severities of depression and anxiety, as well as the headache indices, at all four time points. The correlations of the SF-BP score with depression (correlation coefficient (*r*) ranged from − 070 to − 0.41), anxiety (*r* = − 0.59 to − 0.24), and headache intensity (*r* = − 0.59 to − 0.41) and frequency (*r* = − 0.54 to − 0.33) were negative. The correlations of the PS score with depression (*r* = 0.67 to 0.37), anxiety (*r* = 0.57 to 0.27), and headache intensity (*r* = 0.63 to 0.49) and frequency (*r* = 0.60 to 0.43) were positive.

### Self-reported percentages of time with pain symptoms in the past ten years

The mean (± SD) percentages of time spent suffering pain in the head, neck and/or shoulder, back, and general muscles in the past 10 years were 30.2 ± 28.0, 47.0 ± 31.5, 32.6 ± 30.5, and 29.8 ± 30.8, respectively. The self-reported percentages of time spent with pain in the head (*r* ranged from − 0.27 to − 0.50 for the SF-BP; 0.35 to 0.55 for the PS), neck and/or shoulder (*r =* − 0.29 to − 0.60 for the SF-BP; 0.28 to 0.68 for the PS), back (*r* = − 0.23 to − 0.56 for the SF-BP; 0.35 to 0.67 for the PS), and general muscles (*r* = − 0.28 to − 0.59 for the SF-BP; 0.35 to 0.79 for the PS) were significantly (all *p* <  0.01) correlated with the SF-BP and PS scores at the four time points.

**Fig. 1:**
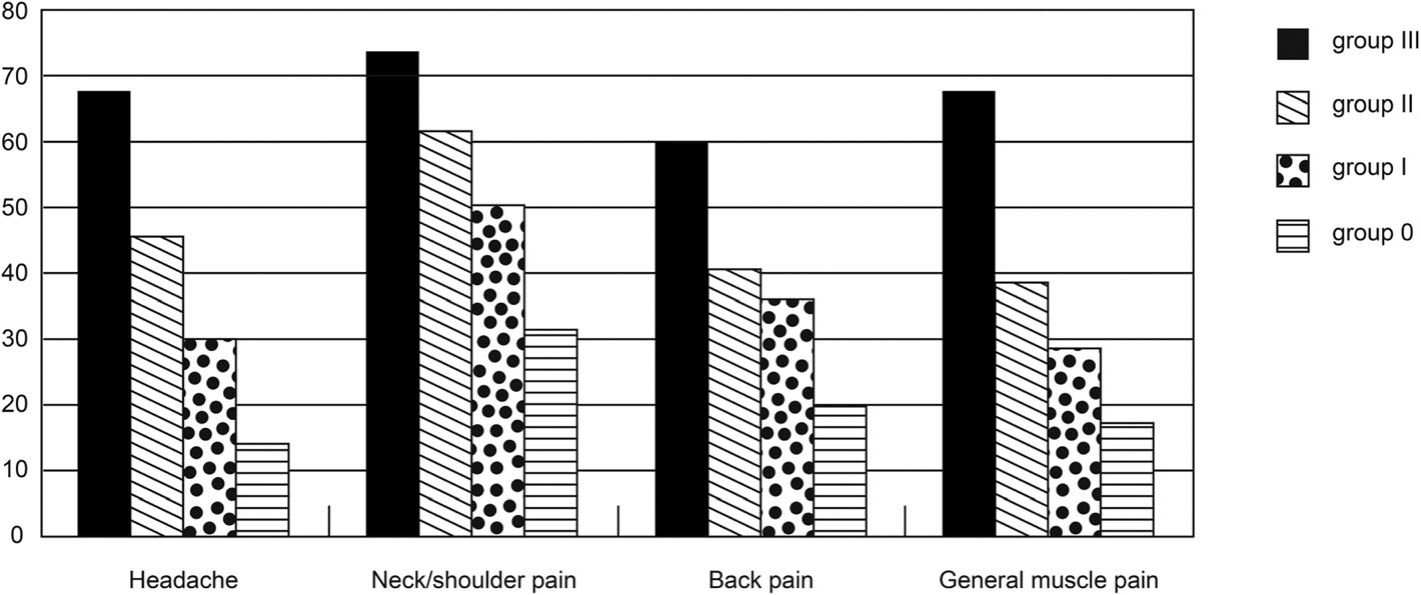
Self-reported percentages of time with pains in the past ten years in different groups. Group III, II, I, and 0 represent patients who had migraine at all, any two, any one, and none of the three time points (including baseline, the two-year point, and the ten-year point), respectively. Significant differences were noted between the four groups in terms of the four pain symptoms

Figure 1 presents the differences in the self-reported percentages of time spent with pains in the different groups. Patients with migraine at the three time points _(B, 2Y, 10Y)_ (group III) spent the highest percentage of time with the four pain symptoms (ranging from 60.0% to 73.7%), followed by patients with migraine at any two of the three points (group II). In contrast, patients without migraine at the three time points (group 0) had the lowest percentage of time spent with pain. In the post hoc analysis, significant differences between groups were noted for headache (group 0 vs. group I, II, and III; group I vs. group III), neck/shoulder pain (group 0 vs. group I, II, and III), back pain (group 0 vs. group II and III) and general muscle pain (group III vs. group 0, I, and II).

### Factors independently associated with pain indices

Migraine was a significant factor associated with the SF-BP and PS scores after controlling for demographic variables, anxiety disorders at baseline, the severities of depression and anxiety at the same time points, and pharmacotherapy (Table 4). Compared with patients without migraine, in patients with migraine, the SF-BP score was lower by 8.93 points and the PS score was higher by 1.33 points after controlling for other factors.

**Table 4:** Independent variables associated with pain indices^a^

Dependent variable	Independent variable	Estimate	Standard error	95% CI	*P*
SF-BP	Migraine (yes vs. no)	−8.93	1.40	−6.20 to −11.67	< 0.001
	HAMD (one-point increment)	−1.20	0.10	−1.00 to − 1.40	< 0.001
	HADS-A (one-point increment)	−0.59	0.19	−0.22 to −0.96	< 0.01
	Educational years (one-year increment)	0.90	0.29	1.47 to 0.32	< 0.01
PS	Migraine (yes vs. no)	1.33	0.23	1.78 to 0.89	< 0.001
	HAMD (one-point increment)	0.19	0.02	0.22 to 0.16	< 0.001
	HADS-A (one-point increment)	0.12	0.03	0.18 to 0.06	< 0.001
	Educational years (one-year increment)	−0.13	0.05	−0.03 to −0.23	0.01
	Married (yes vs. no)	0.53	0.21	0.95–0.12	0.01
	Age (one-year increment)	0.03	0.01	0.05 to 0.004	0.02
	Anxiety disorders (yes vs. no)	0.52	0.24	1.00 to 0.05	0.03

*HAMD* hamilton depression rating scale, *HADS-A* anxiety subscale of the Hospital Anxiety and Depression scale, *SF-BP* the bodily pain subscale of the Short Form 36, *PS* pain subscale of the Depression and Somatic Symptoms scale

^a^Generalized Estimating Equations models were used in this Table

## Discussion

Migraine was a significant factor associated with the two pain indices after controlling for depression, anxiety, pharmacotherapy, and other factors (Table 4). Compared with MDD patients without migraine, MDD patients with migraine had a poorer HRQoL in the pain dimension and a higher pain severity at the four time points (Table 2). Moreover, patients with migraine also had greater severities of depression and anxiety (Table 3). Table 4 shows that migraine, HAMD score, and HADS-A score were independent factors associated with the PS and SF-BP. Therefore, the severities of depression and anxiety could only explain part of the pain symptoms in MDD patients. Migraine is an important but relatively neglected factor associated with pain symptoms, and was found to be independent of the effects of depression and anxiety. In fact, a previous study reported that migraine affects pain symptoms among psychiatric outpatients to a greater degree than MDE [20]. As discussed above, migraine was an important factor associated with pain symptoms in this study (Table 4). Pharmacotherapy for MDD that focuses on depression and anxiety only and neglects the role of migraine in pain symptoms might fail to fully resolve pain symptoms and lead to residual symptoms. This might be one of reasons why pain symptoms often become residual symptoms [38]. Our results implied that depression and migraine should be treated simultaneously. Migraine prevention is important, because migraine is not only associated with pain, but also with cardiovascular diseases, cerebrovascular diseases, and fibromyalgia [15–19]. In an animal study, decreases in pain symptoms were associated with improvement of depressive-like behaviors [39]. Depression and pain symptoms interact. There is a possibility that improving pain symptoms might decrease depressive symptoms among patients with depression; however, more research and evidence are indicated to support this hypothesis. Nonsteroidal anti-inflammatory drugs are commonly-used medications for headache [40]. Patients with MDD who use nonsteroidal anti-inflammatory drugs for headache should be monitored for medication overuse, because depression and anxiety are associated with a higher risk of medication-overuse headache [41, 42]. In fact, some therapeutic strategies for depression are also effective in treating migraine, including antidepressants (such as serotonin-norepinephrine re-uptake inhibitors and amitriptyline), cognitive behavioral therapy, and relaxation techniques [43]. Cognitive behavioral therapy might improve patients’ self-management of diseases and promote adaptive behaviors and skills for coping with stress [43]. Physicians might use multi-model therapeutic strategies based on patient preference, disease severity, possible adverse side effects, and prior adherence history.

Two points are worth noting: 1) The patients in group III had the highest percentage of time spent with pain in the past 10 years (Fig. 1). The higher the frequency of migraine, the more often the patient suffered from pain symptoms. In fact, the patients in group III suffered from pain most of the time during the 10 years (ranging from 60.0% to 73.7%). Therefore, migraine should be treated and prevented during treatment for MDD. 2) For a long time, pain symptoms were considered a part of MDD symptoms [3]. In this study, the severities of depression and anxiety were significantly correlated with the SF-BP and PS scores at the same time points. This demonstrated that pain symptoms were associated with the severities of depression and anxiety. Moreover, our results also showed that pain symptoms in MDD patients might be partially associated with the comorbidity of migraine. Among patients without migraine at all time points (group 0), the self-reported percentage of time with pain was significantly lower than that of the other groups. The causal relationships between depression, anxiety, migraine, and pain symptoms should be further studied.

Migraine resulted in a poorer HRQoL in the pain dimension and a higher pain severity at the long-term follow-up points. This may result from several reasons: 1) Migraine is associated with sensory hypersensitivity [44]. Patients with migraine exhibit generalized pressure pain hypersensitivity in the head as compared with healthy controls [45]. Sensory stimuli can trigger migraine attacks, and central sensitization, characterized by abnormal neuronal excitability, allodynia, and hyperalgesia, may occur when headache attacks are experienced repeatedly [10, 46]. Compared with healthy controls, patients with migraine demonstrated increased neural activity in response to negative emotional stimuli [47]. Therefore, MDD patients with migraine may be more susceptible to other pain symptoms. Central sensitization may be one of the possible mechanisms shared between migraine, pain symptoms, and somatization [48]. 2) The SF-BP and PS include a headache component. Based on the ICH-3 beta criteria, migraine attack is characterized by moderate to severe headache, and is accompanied with physical activity limitation and other somatic symptoms. These may cause functional impairment and significant negative impacts on HRQoL. In fact, migraine was ranked as the seventh leading cause of functional disability [49].

Some limitations or bias should be addressed. 1) Approximately half of the subjects did not attend the ten-year follow-up appointment. Patients who refused to continue to participate in the study or who were unable to be contacted might be associated with a greater severity of depression, which may have caused bias. 2) The interval between the two-year and ten-year follow-up points was long. Unequal follow-up intervals might have caused bias. Frequent follow-up is required in future studies. 3) Due to memory bias, the self-reported percentage of time spent suffering pain in the past 10 years might be not very accurate. Although the self-reported percentages of time with pain were gross estimations, the self-reported indices were significantly correlated with the SF-BP and PS scores at all time points. 4) Although pharmacotherapy was included in the GEE model, the kind and amount of medication were not controlled, because the study was of an observational design. 5) The study excluded MDD patients with other chronic medical diseases at baseline and did not monitor whether these medical diseases and factors related to cardiovascular risk had developed at the follow-up points. In future studies, medical comorbidities should be monitored at follow-up, because these comorbidities might influence the pain threshold [50].

## Conclusion

After controlling for depression, anxiety and other factors, migraine was independently associated with a poorer HRQoL in the pain dimension and more severe pain symptoms among patients with MDD. During the ten-year study period, the higher the frequency of migraine, the more often patients with MDD suffered from pain symptoms. Migraine prevention should be integrated into the treatment of depression, because simultaneous treatment of depression and migraine might help to improve pain symptoms and the score in the pain dimension of the HRQoL. Physicians should employ therapeutic strategies, including pharmacotherapy, cognitive behavioral therapy, and relaxation techniques, for the simultaneous treatment of depression and migraine.

## Abbreviations

DSSS: Depression and Somatic Symptoms Scale
GEE: Generalized Estimating Equations
HADS-A: anxiety subscale of the Hospital Anxiety and Depression Scale
HRQoL: health-related quality of life
ICHD-2: International Classification of Headache Disorders, 2nd edition
ICHD-3 beta: International Classification of Headache Disorders, 3rd edition (beta version)
MA: migraine with aura
MDD: major depressive disorder
MDE: major depressive episode
MO: migraine without aura
PS: pain subscale of the Depression and Somatic Symptoms Scale
SF-BP: bodily pain subscale of the Short Form-36
VAS: visual analog scale

## References

[1] Ligthart L, Gerrits MM, Boomsma DI, Penninx BW (2013). Anxiety and depression are associated with migraine and pain in general: an investigation of the interrelationships. J Pain.

[2] Hung CI, Liu CY, Fu TS (2015). Depression: an important factor associated with disability among patients with chronic low back pain. Int J Psychiatry Med.

[3] Jaracz J, Gattner K, Jaracz K, Górna K (2016). Unexplained painful physical symptoms in patients with major depressive disorder: prevalence, pathophysiology and management. CNS Drugs.

[4] Mcintosh AM, Hall LS, Zeng Y, Adams MJ, Gibson J, Wigmore E (2016). Genetic and environmental risk for chronic pain and the contribution of risk variants for major depressive disorder: a family-based mixed-model analysis. PLoS Med.

[5] Harada E, Satoi Y, Kikuchi T, Watanabe K, Alev L, Mimura M (2016). Residual symptoms in patients with partial versus complete remission of a major depressive disorder episode: patterns of painful physical symptoms in depression. Neuropsychiatr Dis Treat.

[6] Jeon HJ, Woo JM, Kim HJ, Fava M, Mischoulon D, Cho SJ (2016). Gender differences in somatic symptoms and current suicidal risk in outpatients with major depressive disorder. Psychiatry Investig.

[7] Lin HS, Wang FC, Lin CH (2015). Pain affects clinical patterns and treatment outcomes for patients with major depressive disorder taking fluoxetine. J Clin Psychopharmacol.

[8] Novick D, Montgomery W, Aguado J, Peng X, Haro JM (2017). Factors associated with and impact of pain persistence in Asian patients with depression: a 3-month, prospective observational study. Int J Psychiatry Clin Pract.

[9] Novick D, Montgomery W, Moneta MV, Peng X, Brugnoli R, Haro JM (2015). Chinese patients with major depression: do concomitant pain symptoms affect quality of life independently of severity of depression?. Int J Psychiatry Clin Pract.

[10] De Tommaso M, Sciruicchio V (2016). Migraine and central sensitization: clinical features, main comorbidities and therapeutic perspectives. Curr Rheumatol Rev.

[11] Dindo LN, Recober A, Haddad R, Calarge CA (2017). Comorbidity of migraine, major depressive disorder, and generalized anxiety disorder in adolescents and young adults. Int J Behav Med.

[12] Plesh O, Adams SH, Gansky SA (2012). Self-reported comorbid pains in severe headaches or migraines in a US national sample. Headache.

[13] Serafini G, Pompili M, Innamorati M, Gentile G, Borro M, Lamis DA (2012). Gene variants with suicidal risk in a sample of subjects with chronic migraine and affective temperamental dysregulation. Eur Rev Med Pharmacol Sci.

[14] Yang Y, Ligthart L, Terwindt GM, Boomsma DI, Rodriguez-Acevedo AJ, Nyholt DR (2016). Genetic epidemiology of migraine and depression. Cephalalgia.

[15] Mahmoud AN, Mentias A, Elgendy AY, Qazi A, Barakat AF, Saad M (2018). Migraine and the risk of cardiovascular and cerebrovascular events: a meta-analysis of 16 cohort studies including 1152407subjects. BMJ Open.

[16] Tana C, Giamberardino MA, Cipollone F (2017). microRNA profiling in atherosclerosis, diabetes, and migraine. Ann Med.

[17] Giamberardino MA, Affaitati G, Martelletti P, Tana C, Negro A, Lapenna D (2015). Impact of migraine on fibromyalgia symptoms. J Headache Pain.

[18] Tana C, Tafuri E, Tana M, Martelletti P, Negro A, Affaitati G (2013). New insights into the cardiovascular risk of migraine and the role of white matter hyperintensities: is gold all that glitters?. J Headache Pain.

[19] Tana C, Santilli F, Martelletti P, di Vincenzo A, Cipollone F, Davì G (2015). Correlation between migraine severity and cholesterol levels. Pain Pract.

[20] Hung CI, Liu CY, Wang SJ (2013). Migraine predicts physical and pain symptoms among psychiatric outpatients. J Headache Pain.

[21] Oedegaard KJ, Fasmer OB (2005). Is migraine in unipolar depressed patients a bipolar spectrum trait?. J Affect Disord.

[22] Hung CI, Liu CY, Chen CY, Yang CH, Wang SJ (2014). The impacts of migraine and anxiety disorders on painful physical symptoms among patients with major depressive disorder. J Headache Pain.

[23] Hung CI, Liu CY, Yang CH, Wang SJ (2012). The negative impact of migraine on quality of life after four weeks of treatment in patients with major depressive disorder. Psychiatry Clin Neurosci.

[24] Hung CI, Liu CY, Yang CH, Wang SJ (2016). Headache: an important factor associated with muscle soreness/pain at the two-year follow-up point among patients with major depressive disorder. J Headache Pain.

[25] Hung CI, Liu CY, Yang CH, Wang SJ (2015). The impacts of migraine among outpatients with major depressive disorder at a two-year follow-up. PLoS One.

[26] Hölzel L, Härter M, Reese C, Kriston L (2011). Risk factors for chronic depression--a systematic review. J Affect Disord.

[27] American Psychiatric Association (2000). Diagnostic and statistical manual of mental disorders, fourth edition text revision (DSM-IV-TR).

[28] First MB, Spitzer RL, Gibbon M, Williams JBW (2002). Structured clinical interview for DSM-IV-TR Axis I disorders, research version, patient edition (SCID-I/P).

[29] Headache Classification Subcommittee of the International Headache Society (2004). The international classification of headache disorders, 2nd ed. Cephalalgia.

[30] Headache Classification Subcommittee of the International Headache Society (2013). The international classification of headache disorders, 3rd edition (beta version). Cephalalgia.

[31] Hung CI, Weng LJ, Su YJ, Liu CY (2006). Depression and somatic symptoms scale: a new scale with both depression and somatic symptoms emphasized. Psychiatry Clin Neurosci.

[32] Ware JE, Sherboune CD (1992). The MOS 36-item short-form health survey (SF-36). I Conceptual framework and item selection. Med Care.

[33] Tseng HM, Lu JF, Tsai YJ (2003). Assessment of health-related quality of life, II: norming and validation of SF-36 Taiwan version. Taiwan J Public Health.

[34] Chou YH, Lee CP, Liu CY, Hung CI (2017). Construct validity of the depression and somatic symptoms scale: evaluated by Mokken scale analysis. Neuropsychiatr Dis Treat.

[35] Hung CI, Liu CY, Wang SJ, Yao YC, Yang CH (2012). The cut-off points of the depression and somatic symptoms scale and the hospital anxiety and depression scale in detecting non-full remission and a current major depressive episode. Int J Psychiatry Clin Pract.

[36] Hamilton M (1967). Development of a rating scale for primary depressive illness. Br J Soc Clin Psychol.

[37] Zigmond AS, Snaith RP (1983). The hospital anxiety and depression scale. Acta Psychiatr Scand.

[38] Hung CI, Liu CY, Wang SJ, Yang CH (2014). Residual symptoms related to physical and panic symptoms at baseline predict remission of depression at follow-up. Psychopathology.

[39] Zhao X, Wang C, Zhang JF, Liu L, Liu AM, Ma Q (2014). Chronic curcumin treatment normalizes depression-like behaviors in mice with mononeuropathy: involvement of supraspinal serotonergic system and GABAA receptor. Psychopharmacology.

[40] Affaitati G, Martelletti P, Lopopolo M, Tana C, Massimini F, Cipollone F (2017). Use of nonsteroidal anti-inflammatory drugs for symptomatic treatment of episodic headache. Pain Pract.

[41] Lampl C, Thomas H, Tassorelli C, Katsarava Z, Laínez JM, Lantéri-Minet M (2016). Headache, depression and anxiety: associations in the Eurolight project. J Headache Pain.

[42] Sarchielli P, Corbelli I, Messina P, Cupini LM, Bernardi G, Bono G (2016). Psychopathological comorbidities in medication-overuse headache: a multicentre clinical study. Eur J Neurol.

[43] Peck KR, Smitherman TA, Baskin SM (2015). Traditional and alternative treatments for depression: implications for migraine management. Headache.

[44] Demarquay G, Mauguiere F (2016). Central nervous system underpinnings of sensory hypersensitivity in migraine: insights from neuroimaging and electrophysiological studies. Headache.

[45] Baron J, Ruiz M, Palacios-Cena M, Madeleine P, Guerrero ÁL, Arendt-Nielsen L (2017). Differences in topographical pressure pain sensitivity maps of the scalp between patients with migraine and healthy controls. Headache.

[46] De Tommaso M, Sciruicchio V, Delussi M, Vecchio E, Goffredo M, Simeone M (2017). Symptoms of central sensitization and comorbidity for juvenile fibromyalgia in childhood migraine: an observational study in a tertiary headache center. J Headache Pain.

[47] Wilcox SL, Veggeberg R, Lemme J, Hodkinson DJ, Scrivani S, Burstein R (2016). Increased functional activation of limbic brain regions during negative emotional processing in migraine. Front Hum Neurosci.

[48] Grassini S, Nordin S (2017). Comorbidity in migraine with functional somatic syndromes, psychiatric disorders and inflammatory diseases: a matter of central sensitization?. Behav Med.

[49] Steiner TJ, Stovner LJ, Birbeck GL (2013). Migraine: the seventh disabler. J Headache Pain.

[50] Costantini R, Affaitati G, Massimini F, Tana C, Innocenti P, Giamberardino MA (2016). Laparoscopic cholecystectomy for gallbladder calculosis in fibromyalgia patients: impact on musculoskeletal pain, somatic hyperalgesia and central sensitization. PLoS One.

